# The Effect of Malformation and Infection of the Oral Cavity of the Child upon Its Future Health*Read at the Annual Meeting of the Medical Society of the State of New York, Saratoga Springs, May 16, 1916, and published in The New York. State *Journal of Medicine*, February, 1917.

**Published:** 1917-03

**Authors:** Stephen Palmer

**Affiliations:** Poughkeepsie, N. Y.


### THE EFFECT OF MALFORMATION AND INFECTION OF THE ORAL CAVITY OF THE CHILD UPON ITS FUTURE HEALTH.*

BY STEPHEN PALMER, D.D.S., POUGHKEEPSIE, N. Y.

Today is the beginning of a new era in medicine and dentistry when for the first time a member of the specialty of medicine which the speaker represents is by invitation presenting the part dentistry plays in the great field of medicine, and the place it is gaining in the great art of healing, and prophylaxis, and in behalf of the Dental Society of the State of New York and the National Dental Association I thank you for the privilege and honor of appearing here today, not on my personal account, but rather for the profession which I love and ever hope to uplift by my efforts.

* Read at the Annual Meeting of the Medical Society of the State of New York, Saratoga Springs, May 16, 1916, and published in The New York State *Journal of Medicine*, February, 1917.

The dental profession has realized and been preaching for years the importance of a mouth in perfect condition.

We know from observation in our daily vocation the value of a clean and well-kept mouth and teeth. We know that there is nothing that so reduces the vitality of a boy or girl as decayed and aching teeth. We have noted the effect upon the future life of the neglect of the mouth conditions of a child. We know that the pupils in our schools can not do their best unless that portion of their systems, at least, is in a healthy condition. We know that with the mouth and teeth in perfect condition and kept so, many of the bodily ailments and weaknesses can be eliminated, and thus the general health of the children in our schools, and finally the health of the nation will be improved. We know that as the mouth is the gateway of the body, that as the teeth are placed there to perform the first function of the great system of digestion and assimilation, that with them in a perfect, cleanly and healthy condition only can the child be in perfect health, and the future man or woman strong, healthy and intelligent.

The Creator provided each little mouth with twenty teeth, why? Because they were necessary to prepare the food for nourishment of the child body. Let the teeth and mouth of the child between the ages of two and fifteen years be neglected until the teeth are removed to relieve suffering, or left decayed and broken down to the gums so they are of no use, and often abscessed and providing a continual exudation of pus, as well as lodgment for many germs of many of the diseases of the body, during the years when the child is growing and needs every function in perfect order, the child can not masticate properly the food provided, is suffering, is swallowing food unprepared for digestion, and is swallowing bacteria to be assimilated through the system, and you are laying the foundation for a future ill-health that will be carried by them to the grave.

Dr. Victor Vaughan, ex-president of your National Association, says:

"The mouth is the most important port of entry for infection.

"One or more decayed teeth with constant infection so impairs the vitality of the child that physical and intellectual development is impossible.

"Deformity of the jaw and malposition of the teeth interfere with the proper development and function of the brains."

Dr. Osler says, "There is nothing so important to the health of the nation as the hygiene of the mouth," and added to that I make the assertion that there is nothing so important to the health of the coming men and women as the hygiene of the mouth of the boys and girls of today.

Dr. Williams, Superintendent of Bowne Memorial Hospital for Tubercular Patients, says, "Seventy-five per cent. of the children with tuberculosis have very bad teeth and oral infection. When the infections are cleaned up and the teeth restored, the chances for recovery increase, and the return of health is much more rapid."

Dr. Kneph says, "I defy the most skilled physician to either cure or help a tubercular patient who has decayed teeth in the mouth."

We as members of the professions can not ignore these facts as stated by our leading confrere. The sooner we realize the truth of them, and with all our knowledge and effort in diagnosis and treatment, the more satisfactory will be our results, and prophylaxis in medicine by the aid of prophylaxis in dentistry will produce the results unprecedented in their improvement of the health and strength of our fellow citizens.

*Deformities.*—Malformations or deformities of the mouth unless to the extreme are often not noticeable except only to those who have made a study of them, as no two normal human dentures have ever been created exactly alike, in fact it has never been demonstrated that Nature ever duplicated her forms; just so there have never been two cases of malformation exactly alike. Deformity of the teeth which reduces their function, impairs speech, and mars the facial lines are so prevalent that it is now almost a rule rather than an exception. Go where we may, wherever humanity congregates, and we are confronted by those deformities in such numbers that we are amazed.

The reason for the great number of deformities is attributed to the mixture of blood of different races, as we note that in the Grecian and Roman ages when the blood was purely Grecian or Roman deformities were practically unknown.

Dr. Wuerpel says: "The tendency of modern civilization seems to be to create a law for each individual and in face of complex and constantly changing conditions a fixed type as a basis or standard to govern the molding of the human face can not be established, yet discouraging even as this seems, we believe there is a law for determining the best balance of the features or at least the best balance of the mouth with the rest of the features, which artists probably knew nothing of. It is a law so plain and so simple that all can understand, and apply it: that the best balance, the best harmony, the best proportions of the mouth in its relations to the other features require that there shall be the full complement of teeth, and that each tooth shall be made to occupy its normal position."

"It must be remembered by us all that it takes several years for the completion of the building of a human denture. We must remember that all parts of the anatomy are liable to abnormalities in development, as your medical literature bears abundant witness, but that no part is more frequently at variance with the normal in its development than the dental apparatus is evidenced by the fact that malocclusion of the teeth in some form is almost the rule rather than the exception.

"We can better understand the reason of this when we remember that the dental apparatus is not an organ with but a single function, like the eye, or the ear, but that it is a very complex structure, with many functions, into which enter not only the jaw, dental arch and teeth, but the muscles of mastication, the lips, tongue, nasal passages, palate and throat, and that in addition to the function of mastication these are also concerned in the vital function of respiration, and also in speaking, singing, whistling, laughing, crying—in short, in the expression of all the various emotions, the different parts and combinations of parts entering into the performance of these various functions and acts are so intimately associated that even slight inharmony in the growth and development of any one may ultimately involve the whole apparatus, interfering with the normal functions of all, and even producing repulsive deformities, for the influence of these parts on each other is always continuous and progressive toward the maintenance of harmony and normality if normal, and toward increase of inharmony and the abnormal if abnormal" (Angle).

There are various causes for facial and mouth deformities, but time will only allow us to enumerate those that come under the attention of the general medical practitioner, the general surgeon, the rhinologists, and the laryngologist.

The sole object in establishing dental dispensaries, and the oral hygiene movement in our public schools, is to teach our future men and women the value of the teeth, or in fact the value of *a tooth.*

Every tooth of both temporary and permanent dentures has a function to perform, namely, assisting in keeping the full denture in perfect occlusion, as the loss of one deciduous tooth before the allotted time for the permanent one to take its place results in the eruption of the permanent tooth in malocclusion, and the loss of one permanent tooth results in a permanent deformity, which impairs the functions of the whole dental apparatus for all future time, therefore one cause of dental deformity is the loss of *one* tooth. The one way to guard the future welfare of our patients is to insist upon the care of every tooth both temporary and permanent that it may be retained.

Thumb, lip and tongue sucking habits so often formed by children cause many deformities, but if the habit can be broken before the permanent teeth erupt it will reduce the number of cases of malocclusion.

The most serious and constant cause of malocclusion is nasal obstructions, namely, adenoid vegetation. Adenoids being a trouble of childhood, and most active during the growth and development of the denture, namely, before the age of fourteen years, it is very important that the rhinologists and the orthodontist should work together, as it is just as useless for the rhinologists to treat the nasal passages without the assistance of the orthodontist as for the orthodontist to attempt to correct the deformities caused by nasal obstruction without the removal first of the cause by the rhinologist.

Every dental surgeon who makes a study of mouth deformities has noted the effect of mouth breathing upon the future health of the individual, causing as it does the contraction or narrowing of the dental arch, the elevating of the hard palate which causes the obstruction of the nasal passages, the obstruction of the tongue, and finally the impairing of speech, and the function of mastication, and the marring of the symmetry of the face.

May we as members of the great professions of the healing art take into consideration finally that the deformities of the mouth from whatever cause retard the functions of the lips, tongue, cheeks, the nasal passages, the hearing and the speech, which although lessened at the age of fourteen or fifteen, by atrophy (in case of adenoids) the evil effects may last through life, and by uniting our efforts assist in reducing this very serious cause of many future ailments of mankind.

*Infection.*—It has taken Doctors Mayo and Hunter to bring to the attention of the medical profession what the dental surgeon has known and been advocating for years, that mouth infection or oral sepsis play an important part in the health or ill health of the individual. Someone has said, "A child's health is only as good as its teeth."

My only ambition at this time is to impress upon my hearers that as through the mouth passes every substance which enters into the development and strength of the body, health is dependent upon its condition.

During the last few years the origin of many infectious diseases has been traced to conditions of the mouth and teeth. As in medicine so in dentistry the radiograph has become an important agent in diagnosis and by its assistance we have been able to uncover many of our shortcomings and by its assistance we are solving many problems and correcting many errors of the past.

An unclean mouth is one of the causes of many bodily weaknesses and the value of oral prophylaxis has been proven in many cities by the establishment of dental dispensaries, results of which have been that the child is healthier, and many nervous diseases and much retardation is eliminated, and thus the intellectual condition is much improved. The propaganda is to be continued until there will be established in every city and town a dental dispensary.

I have treated exhaustively the subject of malocclusion, as I believe therein lies the origin of many future mouth infections; to malocclusion is attributed the origin of pyorrhea alveolaris: as teeth not in a position that is normal are thus not in use, or if in use are by irregularity receiving undue pressure from an unintended direction are always pyorrhetic, and as pyorrhea is not a disease of childhood (or not so prevalent) we believe that prophylactic precaution, namely early recognition and correction of malocclusion, will reduce the increase of this practically incurable disease. Also irregularity of teeth makes the cleansing of them more difficult, therefore providing lodgment for food and eventually caries.

"A clean tooth never decays," is our slogan, and to that might be added, teeth in correct position or occlusion are easy to clean, and therefore never decay.

Again the mouth conditions of the child affect the future health. By the neglect of the temporary teeth often until proper mastication is impossible, the carious teeth provide lodgment places for filth and bacteria, which is mixed with the food and swallowed, and also many teeth in that neglected condition become abscessed and the exudation of pus is also carried into the system with the food.

If we as fellow practitioners of the different branches of medicine would unite our efforts, by early oral prophylaxis many of the problems which are baffling the medical world would be eliminated.

We have the value of strength demonstrated in the German army of today where they have had compulsory dentistry in many cities, as a child was not allowed to enter school unless the teeth were in order. We have the neglect of teeth demonstrated in the English army (the nation of the whole world that has neglected the teeth and mouth), where many, many men are obliged to return home because of mouth conditions.

In September a law in our state goes into effect which legalizes dental hygienists, a step toward the ideal, we believe, as these trained young ladies may go into our schools and assist in educating as well as applying oral prophylaxis.

By our united efforts I prophesy a healthier, stronger and brighter coming generation.

